# Does Resistance Indicate Malposition? A Standardized Comparison of Pedicle Screw Placement

**DOI:** 10.3390/bioengineering12111254

**Published:** 2025-11-16

**Authors:** Sascha Kurz, Benjamin Fischer, Janine Schultze, Florian Metzner, Toni Wendler, Christoph-Eckhard Heyde, Stefan Schleifenbaum

**Affiliations:** 1 ZESBO—Center for Research on Musculoskeletal Systems, Faculty of Medicine, Leipzig University, 04103 Leipzig, Germany; 2 Department of Orthopaedic, Trauma and Plastic Surgery, University of Leipzig Medical Center, 04103 Leipzig, Germany

**Keywords:** biomechanics, spine, experiment, pedicle screw, mechanical resistance, screw misplacement, malposition, torque

## Abstract

Pedicle screw malpositioning remains a frequent complication, with reported rates from 2% to 15%, often leading to revision surgeries. Analyzing mechanical resistance and torque encountered during screw insertion has been implicated as a promising approach for real-time detection. Five fresh-frozen human thoracolumbar spine specimens were utilized in this study. Using 3D-printed templates, correct trajectories were systematically compared against four defined malpositions (medial, lateral, superior, superolateral), with offsets ranging from 2.0 mm to 3.5 mm. Drilling, tapping, and insertion phases were conducted at a constant speed and defined feed force. Contrary to the anticipated behavior, malpositioned trajectories showed no statistically significant difference in peak torque compared to correct trajectories across all phases (e.g., tapping p=0.944, r=0.01; insertion p=0.693, r=0.05). Regional stratification between thoracic and lumbar spine also failed to yield significant differences. The only statistically significant difference was observed between the correct trajectory and the superolateral malposition during drilling (p=0.038). Under the tested standardized conditions, torque-based mechanical resistance during pedicle screw placement is generally not a reliable and consistent real-time indicator of malposition.

## 1. Introduction

Pedicle screw fixation is a widely accepted technique for posterior spinal stabilization in the treatment of degenerative diseases, traumatic injuries, and spinal deformities [1,2]. Despite its routine use and the availability of advanced intraoperative navigation systems, pedicle screw malpositioning remains a frequent and clinically relevant complication. Reported misplacement rates vary between 2% and 15%, depending on the definition and detection method used [3,4]. These misplacements are among the leading causes of reoperation within the first 30 days after spinal surgery [5], and in pediatric deformity cases, they represent the most common instrumentation-related complication [6].

Malpositioning can occur in various directions—such as medial, lateral, caudal, superior or superolateral—and can result in cortical breaches of varying severity. While some breaches remain asymptomatic, others pose significant risks to neural and vascular structures, particularly in cases involving the medial or inferior pedicle wall [7,8]. Notably, there is considerable variability among surgeons regarding which malpositions warrant revision, with neurological symptoms being the most decisive factor [8].

A systematic framework for evaluating pedicle screw placement was introduced by Gertzbein and Robbins [9], which grades cortical breaches based on their extent and potential neurological impact. Although originally developed for postoperative assessment, this system can be used as a clinical reference point for defining screw misplacement. Current intraoperative strategies to detect malpositioning include fluoroscopy, CT-based navigation, and neuromonitoring. While these methods can reduce the risk of screw misplacement, they are associated with increased radiation exposure, cost, and limited real-time responsiveness [3]. Consequently, there is growing interest in alternative feedback mechanisms that can provide immediate intraoperative information about screw trajectory.

One promising approach involves analyzing the mechanical resistance and torque encountered during screw insertion. These parameters reflect the interaction between screw geometry, bone quality, and trajectory. In ideal insertions, the resistance profile follows a predictable curve; deviations from this pattern may indicate cortical breaches or trajectory errors. Preliminary in vitro studies have implicated that both peak torque and resistance fluctuations differ significantly between correctly placed and malpositioned screws [10,11,12,13].

Other studies postulate a correlation between peak torque and stability, which depends on correct positioning [14], suggesting their potential as real-time indicators of screw alignment. Tai et al. [15] found that screws with a cortical bone trajectory demonstrated significantly higher insertion torque than traditional or modified trajectory screws. This indicates that there are altered resistance curves based on bone engagement patterns. These results suggest that malpositioned screws traversing different bone structures would exhibit characteristic resistance signatures. Peak torque typically occurs when the screw shank passes through the pedicle, approximately at the middle of screw length. Malpositioned screws would theoretically alter this peak timing and magnitude due to Cortical wall breach [16] (sudden resistance loss when violating pedicle walls), bone density variations [17] (different resistance patterns when engaging cortical versus cancellous bone) and angular trajectory changes [18] (modified force vectors affecting torque requirements).

In this study, we systematically investigate the resistance and torque profiles recorded during the drilling, tapping, and insertion phases of pedicle screw placement under controlled laboratory conditions. Using thoracic and lumbar vertebrae from human cadavers, we compare correct trajectories with defined malpositions—medial, lateral, superior, and superolateral—based on parallel displacement from the optimal path. Our aim is to identify mechanical markers that reliably distinguish between accurate and faulty screw placements.

Therefore, the primary research question of this study is whether pedicle screw malpositions could be reliably detected by differences in mechanical resistance during insertion. We hypothesize that malpositioned screws exhibit distinct resistance curves and altered peak torque values compared to correctly aligned insertions. Validating this hypothesis could enable the integration of torque-based feedback into surgical workflows, improving intraoperative decision-making and reducing the incidence of pedicle screw misplacement.

## 2. Materials and Methods

### 2.1. Specimen Acquisition

All body donors provided informed written consent for the donation of their bodies for teaching and research purposes while they were alive. As part of the body donor program regulated by the Saxonian Death and Funeral Act of 1994 (third section, paragraph 18, item 8), the Ethics Committee of the University of Leipzig Medical Center obtained institutional approval for the use of postmortem tissues from the Institute of Anatomy at the University of Leipzig (ethical approval no. 129/21-ck, approval date 1 March 2023). The authors declare that all experiments were conducted according to the principles of the Declaration of Helsinki (as revised in 2013).

Five fresh-frozen human cadaveric thoracolumbar spine specimens (T8–L5) were harvested from donors enrolled in the institutional body-donor program (Table 1). Each specimen comprised five thoracic (T8–T12) and five lumbar vertebrae (L1–L5), yielding ten vertebrae per cadaver. Soft tissue was removed to expose the bony anatomy of the pedicles and vertebral bodies while preserving osseous integrity. After preparation of the spinal segments by the Institute of Anatomy of Leipzig University, the specimens were stored at −80 °C. Prior to the start of the experimental phase, all samples were scanned in a frozen state using quantitative computed tomography (QCT) (Ingenuity CT/Brilliance Big Bore | Koninklijke Philips N.V., Amsterdam, NL-NH) with 1 mm slice thickness and a tube voltage of 120 kV, utilizing a calcium-hydroxyapatite phantom (Bone Density Calibration Phantom 6 H200—QRM-50124|QRM GmbH, Möhrendorf, DE-BY). Mean cancellous Hounsfield units (HU) were measured in Mimics Innovation Suite (Version 24|Materialise NV, Leuven, BE-VBR) and converted to volumetric bone mineral density (vBMD) for each vertebra according to Metzner et al. [19] using linear regression based on the procedure of Brett and Brown [20].

### 2.2. Preliminary Planning

The previously performed CT scans were used to plan pedicle screw trajectories and insertion depths for each vertebral level. Based on Gertzbein and Robbins [9], five trajectory types were defined: correct (c), lateral (l), medial (m), superior (s) and superolateral (sl) (Table 2).

To ensure a systematic and balanced distribution of screw misalignments across vertebral levels and cadavers, an initial planning table was created. The assignment of malpositions followed a structured rotational principle: for each vertebral level, the type of trajectory deviation—such as medial, lateral, superior, or superolateral—was shifted downward by one cadaver in a cyclic manner. This approach ensured that each cadaver received a distinct combination of malpositions throughout the thoracolumbar spine, avoiding clustering or repetition of specific trajectory types.

One vertebra per spinal region (thoracic and lumbar) of each cadaver was instrumented bilaterally with correct trajectories to serve as a reference level. The side on which each malposition was applied—left or right—was determined by randomization using Microsoft Excel (Microsoft 365 MSO, Version 2502, Build 16.0.18526.20416, 64 Bit|Microsoft Corporation, Redmond, US-WA). This introduced variability while maintaining control over the overall distribution of trajectory deviations (Table 3).

Following segmentation of the vertebrae from CT data, the initial trajectory plan was reviewed and adjusted based on individual anatomical characteristics, including structural anomalies and fractures. In cases where the originally assigned malposition was deemed biomechanically unfeasible or anatomically incompatible, the trajectory was modified accordingly. If anatomical exclusion criteria—such as severe fractures or compromised pedicle integrity—prevented safe screw placement altogether, the affected vertebrae were excluded from planning and subsequent testing. This applied to two vertebrae (L1 in Cadaver 1 and L5 in Cadaver 4), resulting in four omitted screw placements prior to template design.

Specimen-specific drilling templates were created to enable precise and reproducible screw placement during the experiment. The templates were designed using CAD software (Rhino 7 SR37|Robert McNeel & Associates, Seattle, US-WA), based on segmented vertebral models derived from CT data. For each vertebra, the entry point and transpedicular trajectory were defined in three-dimensional space, and the corresponding screw diameter was represented as a cylindrical volume within the software environment. Once the trajectory was established, the drill path was integrated into the template design to ensure alignment with the pedicle axis. Stable and anatomically conforming fit to the bone surface was guaranteed by equipping each template with pre-shaped contact surfaces and structural support elements tailored to the individual vertebral geometry. The finalized templates were manufactured using PolyJet (Stratasys J850 DAP|Stratasys Inc., Minnetonka, US-MN) 3D printing from VeroPureWhite (VeroPureWhite|Stratasys Inc., Minnetonka, US-MN) material, resulting in high-resolution, rigid guides suitable for the intended experimental application. The preliminary planning was based on the usage of M.U.S.T. pedicle screws (Medacta International, Castel San Pietro, CH-TI) and associated instruments; screw dimensions were planned per manufacturer specifications and recorded in the dataset. The final screw lengths and diameters listed in Table 4 were assigned to the respective vertebrae.

### 2.3. Specimen Preparation

**Figure 1:**
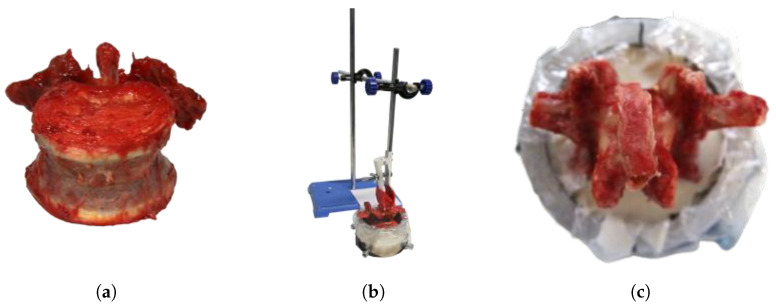
Specimen preparation. (**a**) Separated dissected lumbar vertebra. (**b**) Vertebra in sleeve, held by embedding device. (**c**) Vertebra embedded in rapid casting resin.

Before the tests began, the samples were removed from the ultra-low temperature freezer and gently thawed to 20 °C for 24 h. On the day of testing, all vertebrae were separated and the remaining soft tissue was removed. The vertebrae were then embedded in aluminum sleeves using two-component polyurethane rapid casting resin (RenCast FC 52/53 isocyanate, FC 52 polyol|Huntsman Advanced Materials, East Lansing, US-MI), which was filled with aluminum hydroxide powder (Al(OH)_3_) (Gössl + Pfaff GmbH, Karlskron, DE-BY) (Figure 1). The mass-related mixing ratio of the individual components isocyanate:polyol:aluminum hydroxide was 1:1:3.

### 2.4. Experimental Setup

**Figure 2:**
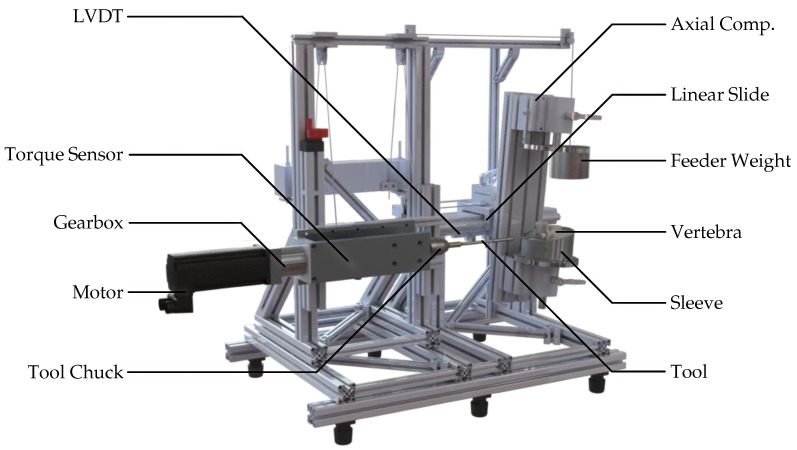
Custom rig for pedicle screw insertion. The specimen and drill-guide template are mounted on a linear carriage; torque and displacement are measured in real time.

A dedicated test rig was developed to enable reproducible biomechanical evaluation of pedicle screw insertion, as shown in Figure 2. The specimen embedding sleeve was securely mounted on a unit for multi-axis rotational compensation, which is coupled to a precision linear slide to ensure reproducible colinear alignment of the insertion axis of all tools with the pre-planned trajectory. To guide the linear slide, a deflected feeder weight was utilized, which applied 27.6 N directly in the slide’s traversing axis.

The average rotational speed of a typical surgical screwdriver was chosen based on the OrthoDrive MBQ-700 motor system (De Soutter Medical Limited, Aston Clinton, GB-BKM). For actuation, a servomotor (Lexium BMH type 0703T01A2A|Schneider Electric GmbH, Düsseldorf, DE-NW) driven by a electric frequency converter (CN6 I/O|Schneider Electric GmbH, Düsseldorf, DE-NW) was coupled to an economy planetary gearbox (PLE060-008-SSSA3AD-R14|Neugart GmbH, Kippenheim, DE-BW). Torque was measured using a pre-amplified slip-ring torque transducer (4501A20HA, 2 Nm to 1000 Nm—valid manufacturer test certificate for measurement uncertainty of 0.1%|Kistler, Winterthur, CH-ZH) and connected to a chuck. A linear variable differential transformer (LVDT) (RACC-50—calibrated in advance of experiments via two-point calibration|MEGATRON Elektronik GmbH & Co. KG, Munich, DE-BY) was used to measure the travel distance resulting from the insertion depth. Signals were amplified by a measurement amplifier (GSV-1A8USB K6D/M16|ME Systeme GmbH, Hennigsdorf, DE-BB) and logged at 1 kHz via the graphical programming environment LabVIEW (Version 2017|National Instruments, Austin, US-TX).

### 2.5. Screw Insertion

Each vertebra, embedded in its aluminum sleeve, was mounted in the test rig. With the feeder weight unloaded, the pedicle axis was precisely aligned using the integrated alignment unit and multi-axis compensation mechanism (see Figure 2), starting with the left pedicle. The pre-manufactured drill-guide template was positioned over the spinous process and aligned with both pedicles using anatomically contoured support elements.

Once alignment was completed, the surgical drill was mounted in the tool chuck. The travel distance of the linear slide was set according to the planned screw length, measured via the integrated caliper system. This value was entered into the test software to calibrate the automatic depth stop, after which the feeder weight was armed and pilot hole drilling initiated. Following tool exchange, the tap was used to cut the thread to full depth. After a final tool change, the pedicle screw was implanted, which was advanced into the prepared tract to the predefined insertion depth.

**Figure 3:**
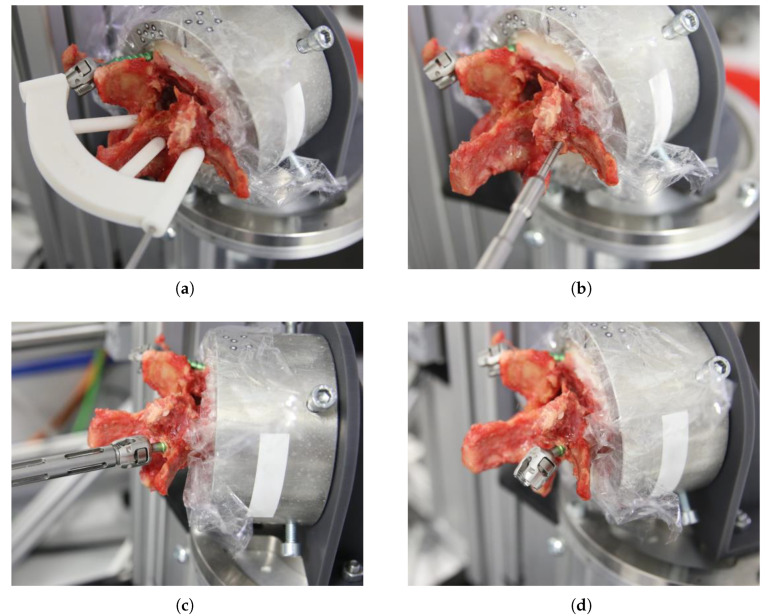
Screw insertion process. (**a**) Drilling the pilot hole using the pre-manufactured drill-guide template. (**b**) Tapping. (**c**) Screw insertion. (**d**) Bileaterally implanted vertebra.

All three procedural steps—drilling, tapping, and screw insertion—were performed at a constant speed of 25 min^−1^, using original instrumentation (Medacta International, Castel San Pietro, CH-TI). The entire insertion process is illustrated exemplarily in Figure 3 on a right pedicle screw. After completion of the left pedicle insertion, the vertebra was repositioned within the test rig to align the right pedicle. The drill-guide template was trimmed around the support shaft of the inserted left screw and the procedure was repeated under identical conditions.

Events that could lead to data corruption were marked with the corresponding error note and excluded from the evaluation. Any other deviations from the planned test procedure were also noted. For example, if the insertion depth was not reached to avoid contact between the screw head and bone, this was noted. Later, the data series was evaluated using the recorded actual measured insertion depth.

### 2.6. Data Acquisition

Torque, insertion depth, and time were continuously recorded during all three procedural phases: drilling, tapping, and screw insertion. Data acquisition was controlled via a custom LabVIEW interface, which also governed motor actuation via the electric frequency converter and sensor synchronization. Upon initiation, the software prompted the user to enter the target rotational speed, the planned travel distance—previously determined via the test rig’s integrated caliper system—and the current procedural step (i.e., drilling, tapping, or screw insertion).

Once confirmed, all connected sensors were automatically zeroed, the offset values were recorded, and real-time recording of torque, time, and displacement was initiated. From these primary signals, feed rate was derived. All four parameters were visualized in synchronized control graphs throughout the procedure. Upon reaching the predefined insertion depth, the measurement was automatically terminated, and the data were exported in .xlsx format using Microsoft Excel.

To ensure traceability and automation during post-processing, a standardized file naming convention was implemented. Each filename encoded the cadaver number, vertebral level and laterality, target revolutions, as well as screw dimensions. This structure ensured that all procedural metadata were embedded directly in the file name, allowing for efficient identification and cross-referencing of measurements across specimens and conditions (see Supplementary Material File S1).

All measurements from the same cadaver were stored within a single file, with separate worksheets created for each procedural step and side. This structure enabled specimen-wise accumulation of data and facilitated subsequent analysis. All data were checked for conclusiveness. Datasets with errors that appeared to result from the data export were excluded from the analysis.

Given the employment of specimen-specific drill-guide templates, experimenters were invariably aware of the trajectory type during screw placement. However, data acquisition was fully automated, so the recorded signals were not influenced by operator knowledge.

Peak torque was determined for each trial by first identifying the three highest individual torque measurements recorded during the respective procedural phase. The three peak values were averaged to yield the top-3-average (top-3-avg), which provides a single, representative measure of maximal torque output for that phase. This metric reduces the influence of transient fluctuations, measurement noise, and isolated artifacts that can occur in a single peak value while capturing the upper performance range of each trial. Prior to calculating the top-3-avg, the torque data were filtered to remove artifactual or mechanically biased segments. For drilling, the distance-referenced data were truncated to exclude the initial and final 2 mm of the trajectory. This eliminated start-up effects when the drill bit first contacted the bone surface and end-stop effects at the completion of the bore. For tapping and screw insertion, only the final 2 mm were excluded to remove end-stop effects. The resulting peak torque (top-3-avg, filtered) values were used as the dependent variable in all subsequent statistical analyses. To ensure objective grouping, the datasets were processed using standardized file names and metadata.

### 2.7. Statistical Methods

Initially, descriptive analyses were performed using box plots in Excel based on the raw data. All subsequent statistical analyses were performed using IBM SPSS Statistics (Version 29|IBM Corporation, Armonk, US-NY). Given the very limited sample size (N=5 cadavers), formal normality testing was not performed due to insufficient statistical power to reliably detect departures from normality. Consequently, non-parametric paired comparisons were conducted using the Wilcoxon signed-rank test for left- and right-sided measurements for each procedural phase (drilling, tapping and screw insertion). Only cadaver-vertebra combinations with complete bilateral data across all three phases were included to ensure direct comparability. All tests were two-sided and exact *p*-values were calculated to account for the small sample size. Effect sizes were expressed as rank-biserial correlations to quantify the magnitude and direction of differences.

To examine whether peak torque differed between correctly positioned (c) and malpositioned (m, l, s, or sl) trajectories, side-specific observations were pooled across all vertebrae (independent of laterality) and compared for each procedural phase (drilling, tapping, and screw insertion). The dependent variable was peak torque (top-3-avg, filtered). Group differences were tested using the two-tailed Mann–Whitney *U* test. Effect sizes *r* were calculated to quantify the magnitude of differences. The enabling of a more detailed assessment of potential differences in peak torque was accomplished by the stratification of the dataset into thoracic (T8–T12) and lumbar (L1–L5) subsets according to vertebral level. All measurements were included within each subset, irrespective of side. Peak torque (top-3-avg, filtered) values were then compared between correctly positioned (c) and malpositioned (m, l, s, sl) trajectories, separately for each procedural phase (drilling, tapping, screw insertion). Group comparisons were performed using the two-tailed Mann–Whitney *U* test, and effect sizes *r* were derived from the standardized test statistic *Z* to quantify the magnitude of the differences.

Furthermore, trajectory-based comparisons were performed to assess whether peak torque differed between correct and malpositioned trajectories. For each procedural phase all available observations were pooled and grouped according to trajectory type: correct (c) versus malpositioned (m, l, s or sl). Due to the distribution and unequal group sizes, group differences were tested using the two-tailed Mann–Whitney U test.

In addition, Spearman’s rank correlation was used to determine whether the top 3-avg values are contingent on the vBMD values that were determined for the respective vertebra side.

## 3. Results

In total, 100 screws were initially planned across all cadavers and vertebral levels, corresponding to 300 possible datasets from drilling, tapping, and insertion phases. After applying exclusion criteria, 226 datasets and 67 placed screws remained for analysis. The distribution of planned versus analyzed screw trajectories is summarized in Table 5, which provides detailed counts per trajectory type.

**Figure 4:**
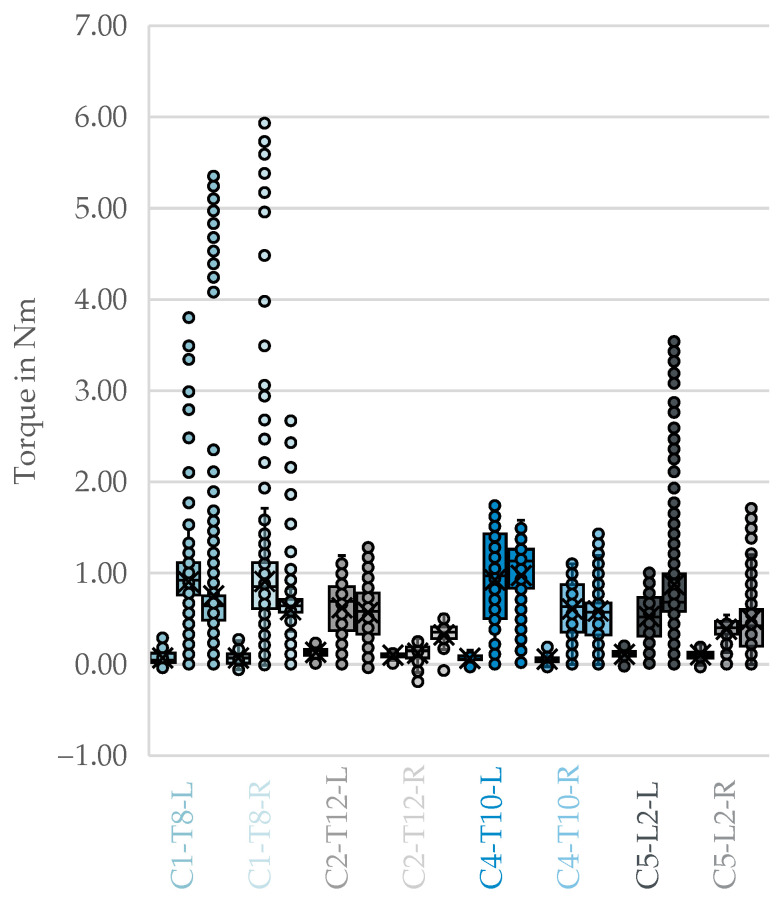
Side-by-side comparison of fully correctly implanted vertebrae containing all phases. From left to right per color: drilling, tapping, and screw insertion with [cadaver]-[vertebral segment]-[side]. X: mean value of the dataset, circles: outliers located more than 1.5 times the interquartile range.

Four cadaver-vertebra combinations with complete bilateral measurements across all three procedural phases were included in the analysis of side-dependence. The box plots in Figure 4 illustrate the three phases of drilling, tapping, and screw insertion for each donor and side. As expected, the tapping and screw insertion phases exhibit higher resistance due to the significantly larger tool and implant diameters.

The results of the Wilcoxon signed-rank test showed that no statistically significant difference was observed in the Drilling phase (Z=−0.552, exact p=0.750). Similarly, in the tapping phase, no significant difference was found (Z=−0.365, exact p=0.875). In the screw insertion phase, values tended to be higher on the left; however, the difference did not reach statistical significance at the 0.05 level (Z=−1.826, exact p=0.125) as shown in Table 6.

**Figure 5:**
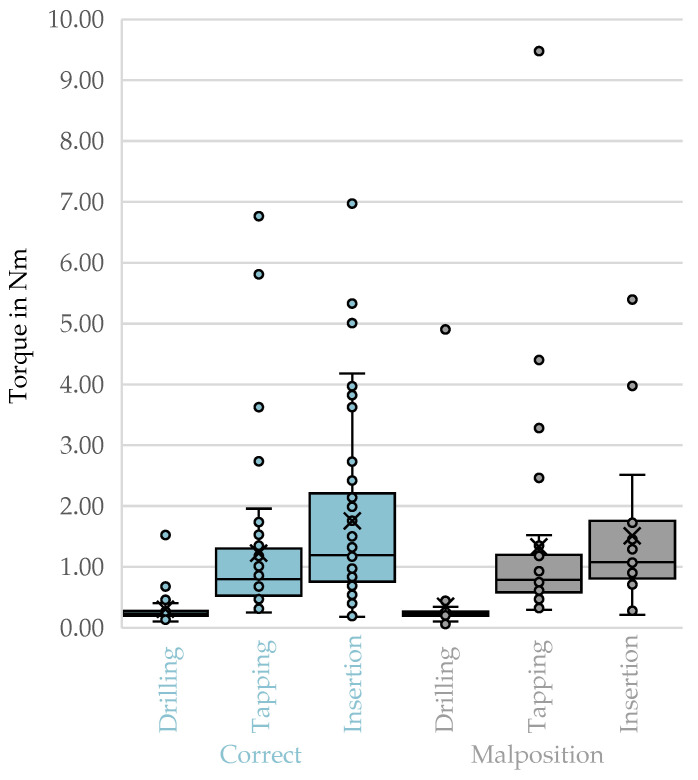
Top-3-avg of the phases of drilling, tapping, and screw insertion for correct trajectories compared to malpositions. X: mean value of the dataset, circles: outliers located more than 1.5 times the interquartile range.

The derived box plots for top-3-avg comparing correct trajectories and malpositions did not show any visible differences Figure 5.

No statistically significant differences in peak torque (top-3-avg, filtered) were observed between correctly and malpositioned trajectories across all vertebrae for any procedural phase (Table 7). For drilling, the Mann–Whitney *U* test yielded U=852.000, Z=−0.714, p=0.475, r=0.08. For tapping, U=609.000, Z=−0.070, p=0.944, r=0.01. For screw insertion, U=494.500, Z=−0.395, p=0.693, r=0.05. All effect sizes were negligible, indicating that the observed differences in rank distributions were not practically relevant.

No statistically significant differences in peak torque for the thoracic subset (T8–T12 upper part of Table 8) between correct and malpositioned trajectories during drilling (U=260.000, Z=−0.022, p=0.983, r=0.00), tapping (U=174.500, Z=−0.575, p=0.566, r=0.09), or screw insertion (U=160.500, Z=−0.227, p=0.820, r=0.04). All observed effect sizes were negligible.

For the lumbar subset (L1–L5, lower part of Table 8), Mann–Whitney *U* tests revealed no significant differences in peak torque for correct versus malpositioned trajectories during drilling (U=172.500, *Z* = −0.908, p=0.364, r=0.14), tapping (U=103.5, *Z* = −0.541, p=0.589, r=0.10), or screw insertion (U=98.500, *Z* = −0.022, p=0.982, r=0.00). All observed effect sizes were negligible to small.

**Figure 6:**
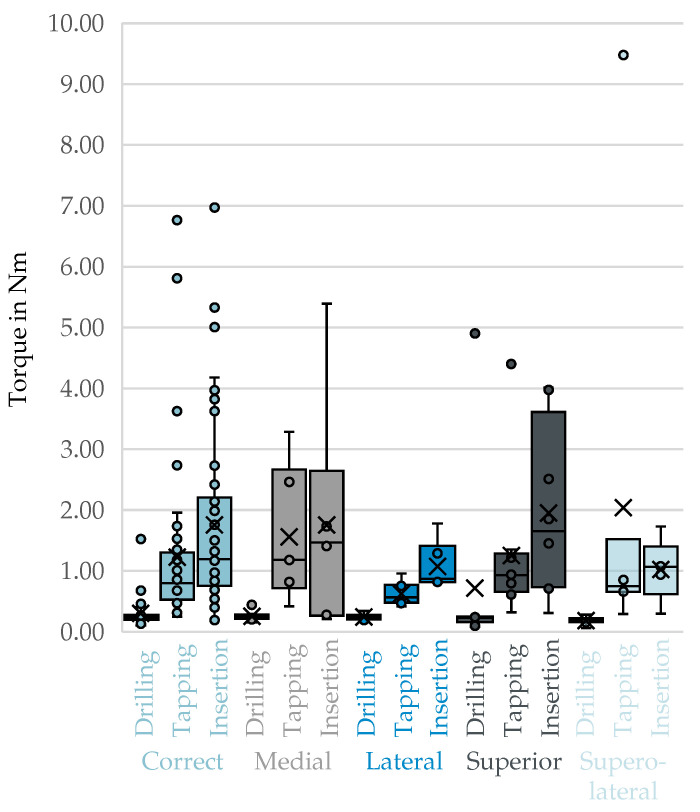
Top-3-avg of the phases drilling, Tapping, and screw insertion for correct trajectories compared to individual malpositions. X: mean value of the dataset, circles: outliers located more than 1.5 times the interquartile range.

No visible differences could be discerned from the box plots of top-3-avg comparing the correct trajectories to the malpositioning during the drilling, thread cutting, and screw insertion phases in Figure 6.

A statistically significant difference in peak torque was observed between the correct trajectory and the superolateral malposition (p=0.038) during the drilling phase. This indicates reduced torque performance in the presence of this deviation. However, all other comparisons within this phase—medial (p=0.605), lateral (p=0.744) and superior (p=0.521)—showed no statistically significant differences (p>0.05). In the tapping phase, none of the comparisons between the correct trajectory and the malpositioned trajectories yielded statistically significant differences in peak torque values (all p>0.05). Similarly, in the screw insertion phase, no significant differences were found between correct placement and any of the tested malpositions (all p>0.05). The detailed results can be found in Table 9 below.

The correlation between top-3-avg and HU values of cortical bone (HU_cb_) revealed a small but non-significant positive association (ρ=0.056, p=0.200, N=226). Similarly, the correlation between top-3-avg and the HU values of spongy bone (HU_sb_) showed a weak, non-significant association (ρ=0.050, p=0.229, N=226) as shown in Table 10.

## 4. Discussion

### 4.1. Background and Objectives

Pedicle screw fixation is a widely used procedure, but malpositioning is common (with rates varying from 2% to 15% [3,4]. Such malpositions are one of the main causes of reoperations within 30 days of spinal surgery and are the most common instrumentation-related complication, particularly in pediatric deformities. According to experimental studies, higher insertion torque and better anchoring stability are achieved when the screws are placed correctly, while malpositioning is associated with reduced insertion torque and a higher risk of micro-movements and screw loosening [21]. This study aimed to identify mechanical markers that could reliably distinguish between correctly aligned and malpositioned screws. This was achieved by systematically examining the resistance and torque profiles during the drilling, thread cutting and screw insertion phases. It was hypothesized that malpositioned screws would exhibit distinct resistance curves and altered peak torque values compared to correctly inserted screws.

### 4.2. Interpretation of the Main Results

Contrary to the central hypothesis, the results showed that there was no statistically significant difference in the measured peak torque (top-3-avg, filtered) between correctly positioned and generally misaligned trajectories (pooled) across all vertebral segments and procedural phases (drilling, tapping and screw insertion). The effects were negligible.

**Phase-related results:** No significant differences in peak torque were found between correctly and malpositioned screws, either in the tapping phase (p=0.944, r=0.01) or in the screw-insertion phase itself (p=0.693, r=0.05). This suggests that, when measured as a top-3 average, peak torque may not be sufficiently sensitive to detect defined cortical breakthroughs (medial, lateral, superior and superolateral) in these later phases.

**Regional analysis:** The stratification of data into thoracic (T8–T12) and lumbar (L1–L5) spinal segments revealed no statistically significant differences in peak torque between correct and misaligned trajectories within individual phases.

**Correlation with screw trajectories:** Even though top-3-avg did not correlate with screw trajectories, this lack of correlation is most plausibly explained by the confounding effects of bone quality and engagement patterns. The nature of the defined malpositions often preserved cancellous bone contact, which also contributed to the lack of correlation. Additionally, while there may be characteristic signal features in the torque/resistance profile, they are likely too small to be detected by the current setup. Consequently, such features could not be reliably measured.

**Correlation with vBMD and HU:** Although the studies by Oh et al. [22] and Walter [21] suggest a correlation between torque and vBMD or HU, we were unable to confirm this in our investigation. This discrepancy may be explained by the fact that we used a controlled, standardized setup, whereas Oh et al. [22] used a surgical screw-in procedure via a conventional posterior approach and freehand technique. Walter [21] also used a machine-based setup but deliberately created screw channels that were 1 mm undersized.

### 4.3. Singular Significant Observation

The study identified only one statistically significant difference: A significant difference in peak torque (p=0.038) was observed when comparing the correct trajectory with the superolateral malposition during drilling. This suggests that deviation in the superolateral direction can result in reduced torque performance. All other specific trajectory comparisons (correct vs. medial, correct vs. lateral, correct vs. superior) during drilling, thread cutting and screw insertion were not significant (all p>0.05).

The fact that only the superolateral malposition showed a difference during drilling may suggest that loss of cortical resistance in the superior/lateral direction during initial pilot hole creation has a more immediate mechanical effect than strictly medial or lateral breakthroughs, which often maintain stronger cortical interactions until final positioning. In addition, the superolateral trajectory combines both angular deviation and cortical wall involvement, which alters the interaction between drill and bone compared to single-axis deviations. This dual deviation could lead to reduced bone stock along the drilling path and earlier cortical breach, resulting in a measurable change in resistance. Furthermore, the superolateral orientation frequently traverses thinner pedicle regions, amplifying local variations in bone density and cortical thickness. These anatomical and geometric factors together may explain why the superolateral malposition produced a distinct resistance signature, whereas other deviations did not. Future studies with larger sample sizes are required to determine whether this effect persists under clinical conditions.

### 4.4. Clinical Implications

The present findings have several important implications for the clinical management of spinal instrumentation during surgery, including error prevention and patient safety. While earlier studies [10,11,12,13] suggested that peak insertion torque may differ between correctly and malpositioned pedicle screws, our results confirm that relying solely on peak insertion torque is insufficient for detecting malpositioned pedicle screws. Since similar peak values can be produced by both correct and incorrect placements [22,23,24,25]. This insight is crucial for intraoperative decision-making as it prevents false reassurance and highlights the need for real-time monitoring of complex torque profiles and fluctuations in order to reliably capture subtle biomechanical changes [22,23,24].

Conversely, evaluating comprehensive torque profiles, including fluctuations, cyclic insertion torque (CIT) patterns and total resistance work (the integral of the torque curve), offers a more sensitive and specific approach to detecting subtle biomechanical changes during screw insertion [26,27,28]. Hee et al. [24] demonstrated that torque profiles differ significantly between cylindrical and conical screws during cortical wall breaches. Conical screws show a continuous increase in torque even after perforation, which can mask malposition events if only peak values are considered. Weidling et al. [28] mathematically demonstrated that the product of outer diameter and insertion torque correlates better with pull-out strength than peak torque alone. Meanwhile, Battula et al. [26] emphasized that optimizing pilot hole size requires consideration of the entire insertion process. Loggia et al. [27] recently described the CIT pattern during robot-assisted screw placement. This method provides both visual and haptic feedback, and may serve as a more reliable indicator of optimal screw trajectory and bone engagement.

The importance of resistance work and integral analysis is further supported by Martin et al. [29], who proposed a simulation method for determining intraoperative torque curves. This enables the development of standardized reference curves for screw insertion. These approaches could facilitate the development of intraoperative guidance systems that provide real-time feedback based on deviations from expected torque profiles [25,27,29]. Furthermore, Martin et al. [29] has proposed that continuous intraoperative torque monitoring could form the basis of machine learning–based predictive models to enhance intraoperative safety and training.

Our findings on side independence are corroborated by robust biomechanical evidence. Oh et al. [22] reported a strong correlation between left and right side torque values in correct insertions; Tai et al. [15] confirmed no significant stiffness differences between sides in controlled biomechanical tests; and Yang et al. [30] validated anatomical symmetry for bilateral pedicle screw fixation. This reproducibility strengthens the case for implementing torque profile analysis as a routine adjunct to navigation and robotic systems [25,27].

From a clinical perspective, these results support the integration of advanced intraoperative assistance systems. Sensor-based instruments, potentially enhanced by machine learning algorithms, could provide real-time feedback on insertion dynamics, including fluctuations, characteristic patterns, and cumulative resistance. This would alert surgeons to critical deviations from expected profiles [27,29]. These approaches align with the broader trend of data-driven, standardized surgical protocols, which improve intraoperative safety, enhance training, and improve quality assurance by providing objective, reproducible biomechanical parameters. Furthermore, integrating cumulative resistance and torque integral values enables personalized therapy planning, including tailoring implant design and pilot hole preparation to specific bone quality [26,28].

Looking ahead, larger, clinically oriented studies are required to validate these concepts under real surgical conditions. These trials should stratify patients by spinal region, bone quality, and deformity type to capture patient-specific variability. Prospective comparisons between conventional insertion and systems enhanced by dynamic profile monitoring would allow for an assessment of not only screw accuracy, but also clinical outcomes such as reoperation rates and neurological safety. Ultimately, establishing standardized reference curves across patient populations may provide the foundation for integration into intraoperative guidance systems and robotic platforms.

### 4.5. Limitations

The results and following conclusions of this study are subject to several limitations:

**Sample size and statistical significance:** Due to the very limited sample size of five human cadavers (N=5), it was not possible to assume a normal distribution. Thus, non-parametric tests were necessary, such as the Wilcoxon signed-rank test and the Mann–Whitney *U* test. The small sample size severely limits the statistical power of the comparisons and increases the likelihood of a type II error, meaning subtle differences may have been overlooked. Although the highly standardized experimental setup and controlled trajectories strengthen internal validity, the generalizability of the findings to broader clinical populations is limited. Larger in vivo studies are required to validate whether the absence of discriminatory power persists under clinical conditions.

**Standardised laboratory conditions:** The experiments were conducted using a special test rig at a constant speed of 25 min^−1^ and a defined feed force of 27.6 N, under highly standardized laboratory conditions. While this ensures repeatability, it does not fully reflect the variable speeds and forces applied during manual or navigation-guided insertion in vivo. In addition, variations in patient-specific anatomy, such as pedicle morphology, bone density, and spinal deformities, can substantially alter torque profiles. Intraoperative conditions, such as limited surgical exposure, bleeding, and soft tissue interference, can also obscure subtle resistance changes. These factors may reduce the detectability of malposition in clinical practice, highlighting the need for future studies under surgical conditions.

**Use of drilling templates:** Specific, 3D-printed drilling templates were used to ensure precise and consistent drilling trajectories. Although these templates could implement the planned positions systematically, they could not eliminate the variability and haptic feedback that would occur in an operating room.

**Geometric definition of misalignment:** Misalignments were defined as parallel displacements (offsets) of between 2.0 and 3.5 mm from the optimal path. Although the Gertzbein and Robbins [9] classification was used as a reference framework, the actual severity of the cortical breakthrough (e.g., the degree of perforation) was not quantified; instead, the mechanical response to the defined offset was examined.

**Signal resolution and detection threshold:** Due to their very small magnitude, potential signal features that could differentiate correct from incorrect trajectories could not be detected. We also examined the complete torque curves, but any characteristics indicating malposition appeared too subtle to be reliably captured with the current setup. Therefore, the statistical analysis was based on a robust, peak-related parameter (top-3-avg), which reduces the influence of transient fluctuations and ensures comparability across specimens.

## 5. Conclusions

This study shows that peak insertion torque alone is not a reliable indicator of pedicle screw malposition during surgery. No consistent or statistically significant differences were observed across all tested deviations and vertebral regions, with the sole exception of reduced torque during superolateral drilling. These findings contradict the initial hypothesis that malpositioning generally alters peak torque values, confirming that mechanical resistance alone lacks discriminatory power. Consistent with previous evidence on screw loosening, our results emphasize that intraoperative decision-making should not rely on peak torque feedback as the primary parameter.

Instead, a comprehensive analysis of the entire torque profile—including cyclic insertion torque patterns, resistance work and integral-based metrics—offers greater sensitivity in detecting subtle biomechanical changes and may provide a more robust foundation for intraoperative guidance systems. However, given the small sample size, the use of highly standardized laboratory conditions, and the geometric definition of misalignment, these conclusions should be regarded as proof of concept. Consequently, future research endeavors should pursue two primary avenues. Firstly, larger in vivo studies are needed to validate whether the absence of discriminatory power for peak torque persists under less predictable clinical conditions; secondly, and perhaps more promisingly, efforts should focus on developing standardized reference curves based on comprehensive torque metrics, which are likely essential for integration into navigation and robotic systems.

## Figures and Tables

**Table 1:** Cadaver data.

ID	Sex	Age in yr	Body Mass in kg	Height in m	BMI in kg m^−2^
1	Male	87	99	1.72	33.5
2	Male	51	61	1.74	20.1
3	Male	83	81	1.72	27.4
4	Male	84	66	1.74	21.8
5	Female	81	82	1.89	23.0

BMI = body mass index.

**Table 2:** Planned trajectory offsets.

Trajectory Misalignment	Deviation Value [9]	Planned Offset
correct (c)	—	0.0 mm
lateral (l)	1–5 mm	3.0 mm
medial (m)	2–5 mm	3.5 mm
superior (s)	1–3 mm	2.0 mm
superolateral (sl)	1–5 mm	3.0 mm

**Table 3:** Initially planned screw malpositions.

Vertebra	Cadaver				
	**1**	**2**	**3**	**4**	**5**
L5	l – R	c – B	s – R	sl – L	m – R
L4	m – L	l – L	c – B	s – R	sl – R
L3	sl – L	m – R	l – L	c – B	s – R
L2	s – R	sl – R	m – L	l – L	c – B
L1	c – B	s – L	sl – R	m – R	l – L
T12	l – R	c – B	s – L	sl – R	m – R
T11	m – R	l – R	c – B	s – R	sl – L
T10	sl – R	m – L	l – R	c – B	s – L
T9	s – R	sl – R	m – L	l – L	c – B
T8	c – B	s – R	sl – R	m – L	l – L

Trajectories: c = correct, l = lateral, m = medial, s = superior, sl = superolateral. Side: L = left, R = right, B = bilateral. Format: [trajectory] – [side].

**Table 4:** Planned screw trajectories and dimensions per vertebra and cadaver.

Vertebra	Cadaver 1			Cadaver 2			Cadaver 3			Cadaver 4			Cadaver 5	
	**L**	**R**		**L**	**R**		**L**	**R**		**L**	**R**		**L**	**R**
L5	sl	c		c	s		c	c		c	l		c	m
	—	—		6/50	6/50		7/55	7/55		7/50	7/50		6/50	6/50
L4	c	s		c	c		l	c		m	c		c	sl
	7/50	7/50		6/50	6/50		7/50	7/50		7/50	7/50		6/50	6/50
L3	c	c		l	c		c	m		sl	c		c	s
	7/50	7/50		6/50	6/50		7/50	7/50		6/50	6/50		6/50	6/50
L2	l	c		m	c		c	sl		c	s		c	c
	7/50	7/50		6/50	6/50		7/45	7/50		6/50	6/50		6/50	6/50
L1	c	m		c	sl		s	c		c	c		l	c
	6/50	6/50		6/50	6/50		6/50	6/50		—	—		6/50	6/50
T12	c	sl		s	c		c	c		c	l		c	m
	5/50	6/50		6/50	6/50		6/50	6/50		6/50	6/50		6/50	6/50
T11	c	s		c	c		c	l		c	m		sl	c
	6/50	6/50		6/50	6/50		6/50	6/50		6/50	6/50		6/50	6/50
T10	c	c		c	l		m	c		c	sl		s	c
	6/45	6/50		6/40	6/50		6/50	6/50		6/50	6/50		5/50	5/50
T9	l	c		m	c		c	sl		c	s		c	c
	5/45	5/50		5/45	5/50		6/50	6/50		5/50	5/50		5/45	5/50
T8	m	c		c	sl		s	c		c	c		l	c
	5/40	5/50		5/40	5/50		5/45	5/50		5/40	5/50		5/50	5/50

Trajectories: c = correct; l = lateral; m = medial; s = superior; sl = superolateral; “—” = not plannable. Side: L = left; R = right. Format: $\frac{\left[\mathrm{trajectory}\right]}{\left[\mathrm{diameter}]/[\mathrm{length}\right]\;\mathrm{in}\;\mathrm{mm}.}$

**Table 5:** Planned versus analyzed screw trajectories (based on Table 3).

Trajectory Type	Planned	Excluded	Analyzed
Correct (c)	60	18	42
Medial (m)	10	4	6
Lateral (l)	10	4	6
Superior (s)	10	2	8
Superolateral (sl)	10	5	5
Total	100	33	67

**Table 6:** Wilcoxon signed-rank test: Side dependence for correct trajectories.

Parameter	Drilling	Tapping	Screw Insertion
*Z*	−0.552 *	−0.365 **	−1.826 **
Asymp. sig. ***	0.581	0.715	0.068
Exact sig. ***	0.750	0.875	0.125

* Based on negative ranks, ** based on positive ranks, *** 2-sided.

**Table 7:** Mann–Whitney *U* test; correctly vs. malpositioned trajectories.

Parameter	Drilling	Tapping	Screw Insertion
*U*	852.000	609.000	494.500
*Z*	−0.714	−0.070	−0.395
Asymp. sig. *	0.475	0.944	0.693

* two-sided.

**Table 8:** Mann–Whitney *U* test; correctly vs. malpositioned trajectories for thoracic and lumbar spine.

Region	Parameter	Drilling	Tapping	Screw Insertion
Thoracic	*U*	260.000	174.000	160.500
	*Z*	−0.022	−0.575	−0.227
	Asymp. sig. *	0.983	0.566	0.820
Lumbar	*U*	172.500	103.500	98.500
	*Z*	−0.908	−0.541	−0.022
	Asymp. sig. *	0.364	0.589	0.982

* two-sided.

**Table 9:** Mann–Whitney *U* test; correct versus malposition per phase.

Phase	Comparison	*N*	Asymp. Sig. *
Drilling	c vs. m	52/10	0.605
	c vs. l	52/9	0.744
	c vs. s	52/9	0.521
	c vs. sl	52/8	0.038
Tapping	c vs. m	41/6	0.232
	c vs. l	41/8	0.137
	c vs. s	41/9	0.649
	c vs. sl	41/7	0.872
Screw insertion	c vs. m	42/6	0.988
	c vs. l	42/6	0.427
	c vs. s	42/8	0.615
	c vs. sl	42/5	0.417

* two-sided. Trajectory classification: c = correct; l = lateral; m = medial; s = superior; sl = superolateral.

**Table 10:** Correlation tests for the dependence of the top-3-avg on derived bone density.

Spearman’s ρ		HU_cb_	HU_sb_
Top-3-avg	Correlation coefficient	0.056	0.050
	Significance *	0.200	0.229
	*N*	226	226

* one-sided, _cb_ cortical bone, _sb_ spongy bone.

## Data Availability

Body donor-related data were anonymized to prevent inferences about their identity. Additional data are available from the corresponding author upon reasonable request.

## Supplementary Materials

The following supporting information can be downloaded at https://www.mdpi.com/article/10.3390/bioengineering12111254/s1: File S1: Experimental data.

## Abbreviations

The following abbreviations are used in this manuscript:

BMI	Body Mass Index
CIT	Cyclic Insertion Torque
CT	Computed Tomography
HU	Hounsfield unit
HU_cb_	Hounsfield unit of cortical bone
HU_sb_	Hounsfield unit of spongy bone
LVDT	Linear Variable Differential Transformer
QCT	Quantitative Computed Tomography
SPSS	Statistical Package for the Social Sciences
vBMD	Volumetric Bone Mineral Density

## Symbols

The following symbols are used in this manuscript:

*N*	Number of observations (or number of pairs for hypothesis tests)
*U*	Mann–Whitney U statistic (rank-based test)
*Z*	Wilcoxon signed-rank statistic (standardized)
